# PCA- and PLSR-Based Machine Learning Model for Prediction of Urea-N Content in Heterogeneous Soils Using Near-Infrared Spectroscopy

**DOI:** 10.3390/s25134176

**Published:** 2025-07-04

**Authors:** Damiano Crescini, Gabriele Mascialino, Nicola Moggia, Giordano Piubeni, Mauro Serpelloni, Emilio Sardini

**Affiliations:** Department of Information Engineering, University of Brescia, Via Branze 38, 25123 Brescia, Italy; gabriele.mascialino@unibs.it (G.M.); nicola.moggia@unibs.it (N.M.); giordano.piubeni@unibs.it (G.P.); mauro.serpelloni@unibs.it (M.S.); emilio.sardini@unibs.it (E.S.)

**Keywords:** partial least squares regression (PLSR), reflectance spectroscopy, sensors, various soil, data models and Urea-N

## Abstract

Determining the soil’s nitrogen supply accurately and quickly is essential for effective agricultural management. This study explores the use of near-infrared (NIR) spectroscopy combined with spectral pre-processing techniques (such as Savitzky–Golay filtering) and partial least squares regression (PLSR) to assess soil nitrogen levels. Six soil types of varying compositions, treated with different levels of Urea-N fertilizer, were examined. Nitrogen-specific NIR peaks were identified, and regression models were consequently developed. Through a comparison of the performance of the models, the most effective model for nitrogen detection was selected. In calibration, the models performed well, with high R^2^ (over 0.9) and low root mean square error (RMSE) values. The second derivative-based (SD) model slightly outperformed the first derivative-based (FD) model in terms of accuracy. Both models showed minimal bias, indicating reliable performance. During validation, the FD model outperformed the SD model in terms of R^2^, root mean square error of prediction (RMSEP), and residual prediction deviation (RPD). Thus, the FD model demonstrated good predictive ability (R^2^ = 0.77, RPD = 2.06), while the SD model was less effective (R^2^ = 0.65, RPD = 1.77). Compared to previous studies, this study uniquely combines real-time online detection capability with low computational cost, unlike most prior offline approaches, and includes model validation across various soil types. Overall, NIR spectroscopy coupled with multivariate models proves to be a promising tool for the detection of nitrogen levels in various soils.

## 1. Introduction

Soil is a vital non-renewable resource that supports food production, climate regulation, and ecosystem services. Its health is fundamental to environmental sustainability and human well-being. However, according to recent estimates, between 60% and 70% of soils in the European Union are currently in an unhealthy state [1]. In response to this alarming trend, the European Commission has introduced a Soil Monitoring Law to promote the assessment and protection of key soil properties [2]. Among these properties, nitrogen (N) plays a central role. As a major determinant of soil fertility, nitrogen is essential for plant development, influencing root growth, foliage density, and crop yield. Accurate and timely assessment of soil nitrogen content is therefore critical for informed agricultural decision-making and the sustainable use of fertilizers. Traditional laboratory-based methods, such as the Kjeldahl digestion technique [3], the indophenol blue colorimetric method [4], and various mineralizable nitrogen tests [5], are widely used and well-established. These techniques, however, are time-consuming, require intensive sample preparation, and depend on access to specialized laboratory equipment.

In recent years, the demand for rapid, non-destructive, and cost-effective alternatives has driven interest in sensor-based approaches for soil analysis. Techniques such as microfluidic detection systems [6], microwave sensors [7], and wireless sensor networks [8] have expanded the toolbox for in situ nutrient monitoring. Among these, visible and near-infrared (Vis-NIR) reflectance spectroscopy has shown significant promise for assessing a wide range of soil properties, including nitrogen content [9]. This technique exploits the interaction of electromagnetic radiation with soil constituents to infer chemical composition based on spectral responses. When paired with advanced multivariate regression methods—such as partial least squares regression (PLSR)—NIR spectroscopy enables the development of predictive models capable of estimating nutrient concentrations from raw spectral data [10,11]. Despite its growing popularity, current research in this area often suffers from important limitations. Many studies are confined to a narrow range of soil types or regional conditions, which constrains the generalizability of the resulting models [12]. Furthermore, the accuracy of NIR-based predictions can be affected by soil moisture, organic matter content, and texture. Model performance can also be challenged by the presence of outliers and the risk of overfitting, especially when working with large or heterogeneous datasets.

The main objective of this study is to evaluate the effectiveness of near-infrared (NIR) spectroscopy for detecting and quantifying urea-derived nitrogen (Urea-N) across six different soil types collected from two continents. These include clayey, sandy, loamy, alluvial, and volcanic soils, which together represent a broad spectrum of global soil classes [13]. Rather than aiming for absolute quantification, this work focuses on assessing the reliability, speed (including inline potential), and cost-efficiency of a spectroscopic approach that does not require wet chemistry or sample extraction. This research integrates three novel elements: (i) the use of a limited-range spectrometer operating in the 1100–1700 nm range; (ii) the application of robust data pre-processing and modeling strategies, including Savitzky–Golay smoothing, principal component analysis (PCA), and PLSR; and (iii) the analysis of a large and diverse soil dataset. Together, these aspects aim to advance the development of practical, accurate, and scalable methods for soil nitrogen estimation, positioning NIR spectroscopy as a viable alternative to conventional laboratory techniques.

## 2. Materials and Methods

### 2.1. Methodological Overview

**Figure 1:**
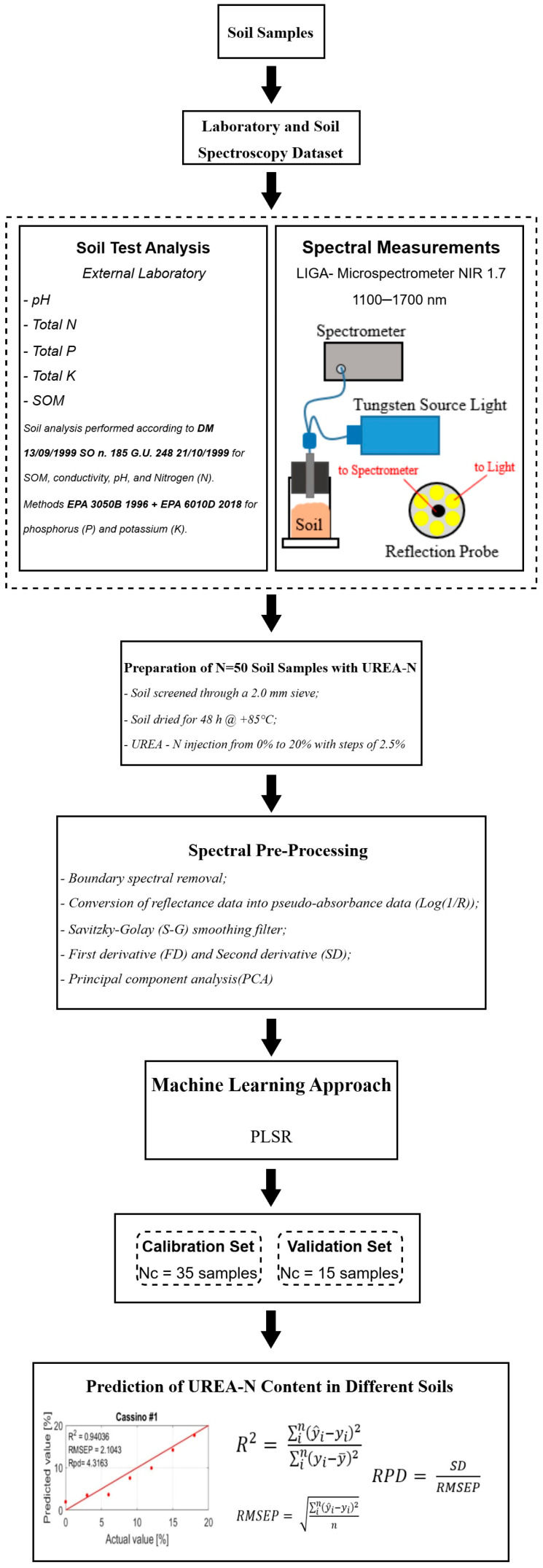
Overall technical flowchart for Urea-N prediction using near-infrared (NIR) spectroscopy.

Figure 1 schematically presents the constituent blocks of the methodology used in this research. Traditionally, soil scientists have relied on lengthy, complex, and often destructive laboratory methods involving toxic chemicals to assess soil characteristics. In contrast, this study employed NIR spectra obtained from soil samples to predict these properties. In detail, the following steps were taken:

(i) Sample Collection

**Figure 2:**
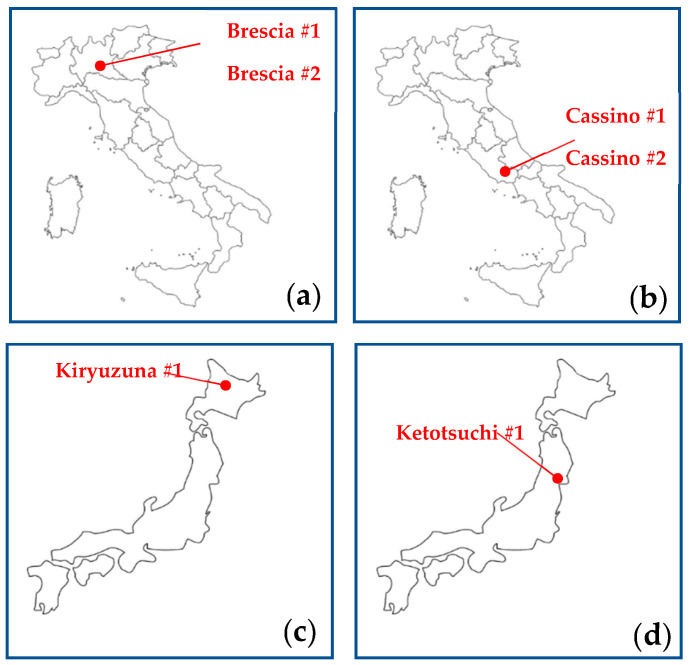
The research region: (**a**) map of Italy—northern sampling area; (**b**) map of Italy—southern sampling area; (**c**) map of Japan—northern area of origin; (**d**) map of Japan—central area of origin.

Soil samples were collected in triplicate from each site (sampling depth: 30 cm ± 5 cm; locations indicated in Figure 2) to ensure both spatial and statistical representativeness of the dataset.

(ii) Laboratory and Soil Spectroscopy Dataset

The collected soils were divided into two batches. The first batch consisted of samples to be sent to an accredited external laboratory for analyses of pH, electrical conductivity (at 20 °C), available nitrogen, available potassium, available phosphorus, and soil organic matter (SOM). The second batch underwent NIR spectroscopy tests using the method presented in this research.

(iii) Sample Preparation and Urea-N Injection

Soils were pre-treated mechanically and thermally. Urea-N injection was performed in succession at different levels of contamination.

(iv) NIR Spectroscopy Analysis

Spectra were extracted from the soils using reflection spectroscopy. During this phase, outlier handling involved analyzing the spectra and re-acquiring them if significant variations in reflectance were observed after the addition of Urea-N.

(v) Data Pre-processing

The spectra were pre-processed and a linear dimensionality reduction technique was applied (principal component analysis, PCA).

(vi) PLSR Modeling and Dataset Partition

The spectra extracted from the soils were divided into samples for calibration and validation of the models; in particular, a statistical method (SPXY) was employed for division of the samples. The PLSR approach was used to model the relationships between independent variables (predictors) and dependent variables (responses).

(vii) Prediction

The PLSR model was applied to evaluate the Urea-N content in the soil.

### 2.2. Soil Sampling and Sample Preparation

The soil assessment was conducted throughout 2023 and 2024 across multiple regions. Soils representing six distinct texture classes were collected as follows: Italian soils were sampled to a depth of 50 cm using a specialized soil-drilling system, while Japanese soils were obtained as commercially available volcanic products purchased online from certified suppliers, and are publicly accessible. Figure 2 and Table 1 show the sampling locations (Italy and Japan) and the corresponding texture classes. For each texture class, eight or nine soil–urea mixtures were prepared. The soil and urea were weighed using a “Kern EWJ 30” (Ziegelei 1, Balingen, Germany) scale, with a resolution of 0.01 g and an accuracy of ±0.05 g. Each sample was prepared using the following procedure:

1. The soil was passed through a 2.0 mm sieve, dried at 85 °C for 48 h, and then weighed.
2. Granular urea was ground into powder using a pestle and mortar, then weighed.
3. The urea was added to the soil and mixed thoroughly until no lumps remained.

This preparation method allowed for the deliberate adjustment of the Urea-N concentration in each sample. Using this technique, the worst-case uncertainty in the Urea-N content was approximately 0.17%, assuming an ideal mixture of the two components and applying uncertainty propagation for division. The uncertainty in the percentage of urea can be calculated as follows:

$$∆R=R\sqrt{{\left(\frac{∆A}{A}\right)}^{2}+{\left(\frac{∆B}{B}\right)}^{2}}$$

where *R* is the ratio between the quantity of the urea and the quantity of the soil; *A* is the quantity of urea and *B* is the quantity of soil; and Δ*A* and Δ*B* are the uncertainties introduced by the scale.

All dosages were calibrated to avoid premature saturation (in some soils, approximately 30% by weight of the soil’s dry mass, corresponding to 100% reflectance) and to prevent variations too subtle to be reliably detected by the instrument.

To ensure homogeneity, water was added to the sieved and dried soils after mixing with the specified Urea-N percentages. The samples were then left to rest for seven days, allowing the Urea-N to distribute uniformly throughout the soil. This homogenization procedure, commonly adopted in soil studies to ensure uniform treatment of samples, was followed by a controlled drying process before spectral measurements were carried out. Table 2 presents the distribution of the samples, along with their corresponding Urea-N concentrations and soil types. A maximum Urea-N concentration of 20% was used. Importantly, the Urea-N concentrations in this study were not obtained through laboratory chemical analysis, but were instead calculated based on known proportions of pure urea powder added to dried, sieved soils. These nominal concentrations, based on mass, were used as reference values for model calibration and validation, as similarly performed in previous works such as those by Tan et al. (2022) [Random Forest model on known N additions] [14] and Nawar et al. (2016) [mass-based reference values for SOM] [15].

The sample size aligns with precedents in the recent literature. For instance, Nawar et al. (2016) [15] used 75 samples to model soil organic matter with PLSR, while Tan et al. (2022) [14] used 43 samples for soil nitrogen detection using NIR and Random Forest. Likewise, Munawar et al. (2020) [16] reported robust model performance with 40 samples using PCR and PLSR. These studies collectively indicate that datasets in the range of 40–75 samples are sufficient to capture spectral variability and enable reliable chemometric modeling. Accordingly, the 50 samples used in this study provided adequate statistical power for the development and validation of models predicting Urea-N in soil.

### 2.3. Examination of Soil Composition Properties

Given the complex nature of soil, both the organic material and water within it respond to NIR spectral patterns. Before examining the optical properties of soils containing different amounts of nitrogen, the key constituent values of the gathered soil samples were measured and assessed. As depicted in Table 3, the compositions of the soils were characterized as follows:

(i) Brescia #1 and Ketotsuchi #1 were acidic soils (pH around 5);
(ii) Brescia #2 was an alkaline soil (pH around 8);
(iii) The electrical conductivity of the soils (a parameter that indicates higher levels of soluble salts or ions) varied significantly, from 90 to 2200 μS/cm;
(iv) The available potassium was much lower in Kiryuzuna #1 (123 mg/kg) than in Brescia #1 (13,900 mg/kg);
(v) The available nitrogen in Ketotsuchi #1 (19.2 g/kg) was higher than in the other soils;
(vi) The organic matter content in Cassino #2 and Kiryuzuna #1 (<1.1 g/kg) was lower than in the other soils.

In general, the chemical compositions of the six soils were very different. This difference between the soils allowed us to validate the PLSR mathematical model under highly heterogeneous conditions. Although the testing laboratory did not explicitly provide the associated uncertainty for all the measured values, the results in Table 3 were obtained from an accredited laboratory operating in compliance with [17].

According to this standard and based on values for which uncertainty was reported (e.g., electrical conductivity), it is reasonable to estimate a typical expanded uncertainty (U) of approximately ±5%, calculated with a coverage factor *k* between 2 and 2.57 at a 95% confidence level. This indicative uncertainty applies to most of the chemical parameters, unless otherwise specified.

### 2.4. Examination of Soil Spectral Characteristics

**Figure 3:**
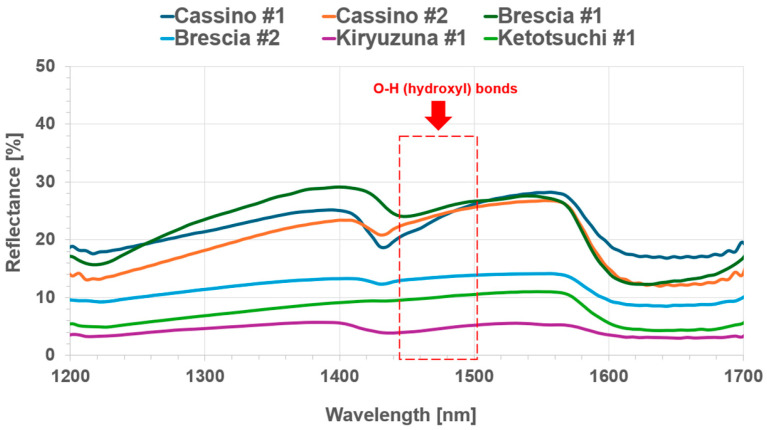
NIR spectra of the six soil samples (Urea-N = 0%).

Figure 3 illustrates the spectral reflectance patterns of the six soils in the NIR region. It is notable that all soil samples exhibit absorption peaks near 1400 nm, which can be attributed to the first overtones of O-H (hydroxyl) bonds. This indicates the presence of water or organic matter in the soils, as these peaks are characteristic of molecular vibrations related to hydroxyl groups. In some spectra, the O-H peak is more evident, which can be attributed to the presence of water within the mineral content, as confirmed by the previous literature [18].

**Figure 4:**
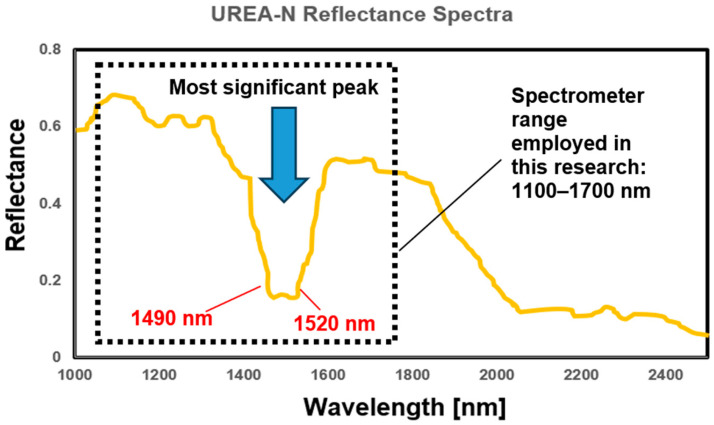
Spectra of standard Urea-N in the range from 1100 to 2500 nm [15].

Figure 4 shows the NIR spectral reflectance curves of standard Urea-N, extracted and magnified between 1100 and 2500 nm according to the NIST Chemistry WebBook, SRD 69 [19]. The most significant peaks in the NIR spectra of Urea-N lie between 1100 and 1700 nm, with a strong absorption peak at around 1500 nm. The absorption decreases as the wavelength increases past 1700 nm, while the region from 1700 to 2500 nm shows weak and less relevant peaks for Urea-N analysis.

Multiple distinct absorption peaks can be observed in the spectral data. According to the relevant literature [20], Urea-N (CH_4_N_2_O) consists of two amine groups (–NH_2_) attached to a central carbonyl group (C=O). In NIR spectroscopy, the fundamental vibrational modes of the N-H bond, particularly relating to bending and stretching vibration, typically appear in the mid-IR region (around 3300–3400 cm^−1^). However, in the NIR range (1100 nm to 2500 nm), these fundamental vibrations give rise to overtones and combination bands, which are weaker but still observable. The absorption peaks at 1490 nm and 1520 nm are likely due to the first overtone of the N-H bending vibration and a combination of N-H stretching and bending modes. The peak at 1490 nm is most commonly associated with the overtone of N-H bending vibration, while 1520 nm corresponds to a combination band involving both N-H bending and stretching vibrations. These spectral features are useful for identifying and quantifying Urea-N in various environments, including agricultural soils. In soil, Urea-N interacts with several components that could affect its NIR spectrum. Water is one of the primary contributors to spectral interference in this range, particularly due to its strong absorption bands around 1450 nm (O-H bending vibration) and 1900 nm (O-H stretching overtone). These bands may overlap with the Urea-N peaks, especially at 1490 nm, complicating the interpretation of the spectra. Organic matter, such as proteins or humic substances, also exhibits N-H stretching and bending features in the NIR range, potentially overlapping with the absorption peaks of Urea-N. Additionally, certain minerals in the soil, especially those with hydroxyl groups (e.g., clays), can produce their own spectral features in the same wavelength region, adding another layer of complexity to the spectral analysis of Urea-N in soil. Therefore, careful spectral deconvolution and data interpretation are required to differentiate the contributions of Urea-N from those of other soil components.

In this research, the focus is exclusively on the analysis of Urea-N in different types of soils under dry conditions and with limited levels of organic matter.

The absorption peaks at 1490 nm and 1520 nm are likely due to the first overtone of the N-H bending vibration and a combination of N-H stretching and bending modes. The peak at 1490 nm (FWHM ≈ 10 nm) and that at 1520 nm (FWHM ≈ 12 nm) were quantified by integrating the absorbance over a ±5 nm window around each maximum, thus minimizing spectral noise and standardizing the calculation of peak area.

The spectrometer used (LIGA-Microspectrometer System NIR 1.7, STEAG microParts GmbH, Dortmund, Germany) operates within a range of 1100 nm to 1700 nm; therefore, the analysis was limited to this specific portion of the spectrum. The spectrometer is based on 128 elements, including InGaAs detectors with a spatial resolution of about 4.5 nm. The objective was, therefore, to focus on the peaks of the spectrum between 1490 and 1520 nm, highlighting their effects on both the first and second derivatives.

### 2.5. Measurement System

**Figure 5:**
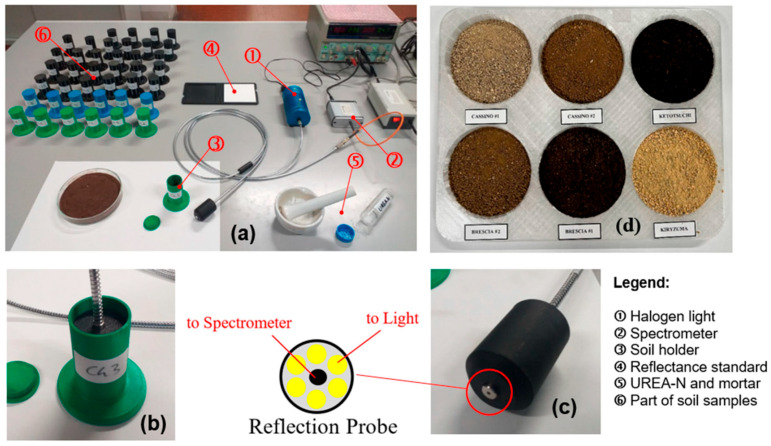
Example of measurement system: (**a**) overview of set-up; (**b**) close-up of soil container with inserted reflectivity probe; (**c**) view of reflectivity probe and detail of optical fibers; and (**d**) view of six soils under study.

Figure 5a–d present, respectively, an overview of the used measurement system, along with its components and a legend; the spectrometric measurement head inserted into the test sample; a detailed view of the reflection probe, showing the morphology of the optical fibers; and a view of the six soils under study. The process for creating soil samples contaminated by the presence of Urea-N was designed to reflect its actual occurrence in natural soils as closely as possible.

Each soil sample was positioned within a circular capsule measuring 3 cm in diameter and 7 cm in depth, ensuring it was level with respect to the spectrometer head’s holder. The samples were illuminated using an HL-2000 20 W tungsten halogen lamp (Ocean Optics, 3500 Quadrangle Blvd, Orlando, FL, USA), and measurements were taken. Radiance was converted to spectral reflectance by dividing the radiance reflected from the soil by that of a standard white reference plate (Spectralon^®^) measured under identical illumination conditions. To minimize instrumental noise, four measurements were averaged for each sample.

**Figure 6:**
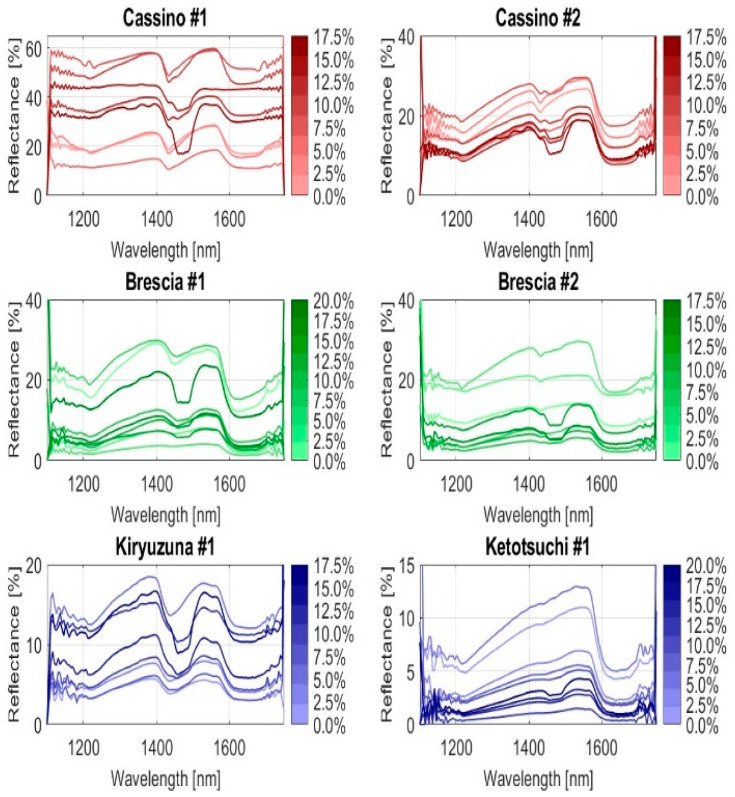
Spectra of the six soils with different Urea-N concentrations.

Figure 6 illustrates the trends of the absorption curves for the six soil samples based on the percentage of Urea-N present. The absorption values and their corresponding peaks can be compared to the trend in Figure 4, which is typical of pure Urea-N at 100%. The main wavelengths (1490 nm and 1520 nm), around which the prediction algorithms of this research were designed to operate, are evident.

A close examination of the trends in the curves reveals that as the nitrogen concentration varies, the reflectance curve changes accordingly. This demonstrates a correlation between Urea-N concentration and light absorption in the soil at specific wavelengths. Based on the trend and morphology of the curves, it can be observed that the NIR spectra display clear and distinct spectral responses corresponding to different Urea-N concentrations in the soils.

Given the significant noise present at both the beginning and end of the spectra, the bands within the range of 1350 to 1645 nm were chosen for data reduction and further examination. This selection was based on their high signal-to-noise ratio, which ensured more reliable and accurate data for subsequent analysis.

### 2.6. IR Spectral Processing and Sample Set Partitioning

Spectroscopy plays a pivotal role in various scientific and industrial applications, enabling the analysis of materials based on their interaction with electromagnetic radiation. However, raw spectral data often suffer from noise, baseline drift, and other artifacts that can hinder accurate interpretation and modeling. A common approach to tackling these challenges involves employing pre-processing techniques to improve the quality of the data and facilitate the extraction of relevant information [21]. A literature review was conducted to identify the most effective pre-processing method for the model, leading to the selection of the Savitzky–Golay (SG) filter and PCA. In refs. [22,23], the impacts of various spectral pre-processing methods on soil property prediction using machine learning algorithms are discussed, including a comparison with techniques such as continuum removal (CR) and multiplicative scatter correction (MSC). The SG filter and PCA are preferred over CR and MSC in NIR spectroscopy as the SG filter effectively smooths data while preserving important spectral features, reducing noise without distorting the signal; meanwhile, PCA, through dimensionality reduction, helps to identify the most significant features capturing variance in the data, enhancing model efficiency and accuracy. In contrast, CR may distort key spectral features through the removal of baseline signals, while MSC primarily corrects for scatter effects, and may not capture complex spectral patterns. Together, the SG filter and PCA improve the signal quality and extracted features, leading to better prediction accuracy. To improve the signal quality and reduce noise, researchers commonly employ the following techniques:

- First-derivative spectra with SG smoothing (FD-SG)—this method enhances the peak detection performance by applying the SG filter to the first derivative of the spectrum.
- Second-derivative spectra with SG smoothing (SD-SG)—similarly to FD-SG, this technique focuses on fine features by applying the SG filter to the second derivative of the spectra.
- PCA—this method is useful for reducing the dimensionality of the problem by projecting the spectra into a different space, where each spectrum is represented as a single point.

For PCA, 20 principal components were retained after examining the scree plot and cumulative explained variance, which accounted for over 95% of the total spectral variance.

These pre-processing steps play a crucial role in optimizing spectral data for subsequent analysis [19].

The formula for SG filtering used in this research is presented in Equation (1):

(1) $$Y_{j}^{*}=\frac{\sum_{i=-m}^{m}C_{i}Y_{j}}{N},$$

where

*Y_j_^∗^* denotes the reconstructed spectral data;

*C_i_* is a filtering coefficient;

*Y_j_* denotes the original spectral data;

*N* is the number of datapoints in the sliding window (*N* = 2*m* + 1);

2*m* + 1 is the window width.

In practice, SG filtering is typically associated with two main parameters: the first relates to the width of the filter window, while the second is usually linked to the polynomial order of the filtering processes. The width of the filter window can influence the smoothing results, where a larger width leads to a smoother spectrum. Similarly, the polynomial order of the fit influences the filtering outcomes [24], with a higher order yielding a smoother fit. For this study, the SG filter window width was set to 11, and the polynomial order was set to 2, as these parameters provide an optimal balance between noise reduction and the preservation of important spectral features. A window width of 11 ensures sufficient smoothing to reduce high-frequency noise while avoiding excessive smoothing that could distort the spectral characteristics. A polynomial order of 2 strikes a balance between fitting the data effectively without overfitting, allowing the method to capture the underlying spectral patterns while minimizing distortion.

The benefits of the abovementioned approaches are as follows:

- Enhanced peak resolution—peaks become sharper and easier to identify.
- Suppressed baseline variations—noise and baseline drift are minimized.
- Improved subsequent data analysis—FD-SG pre-processed spectra are more amenable to chemometric modeling.

In this study, both methods were implemented, and their results were compared. The choice of multivariate statistical approaches and filters significantly impacts the calibration technique used in spectral data analysis [25].

Spectral calibration was performed using MATLAB 9.14, a high-level environment widely used in NIR spectroscopy and chemometric analysis for its advanced tools in signal processing, machine learning, and statistical modeling. In this study, MATLAB 9.14 was used to develop predictive models based on NIR spectra (1100–1750 nm) and their corresponding reference data.

The dataset (*N* = 50) was partitioned into a calibration set, comprising approximately 70% of the data (*N* = 35), and a validation set, which accounted for nearly 30% of the data (*N* = 15). The calibration dataset underwent analysis to determine the optimal pre-treatment options for predicting spectral wavelengths using PLSR. The division of soil samples is crucial when analyzing the compositions of complex solutions that involve spectroscopic measurement processes.

When dealing with a limited number of samples, choosing a calibration set for the developed model that has adequate robustness and universality is essential. In light of this, it was decided that the soil spectrum samples would be partitioned using the x–y distance process described by Galvão et al. [26]. The SPXY algorithm is an extension of the classic Kennard–Stone (KS) algorithm, designed to select a representative subset of samples from a larger dataset. While the KS algorithm only considers the distribution of samples in the x-space (instrumental response), SPXY incorporates both x-space and y-space (dependent variable) distances to ensure a balanced selection. The SPXY algorithm normalizes the Euclidean distances in both spaces, combining them into a single metric, dxy(p,q), which represents the total distance between samples p and q. Like the KS algorithm, SPXY follows a stepwise procedure, iteratively selecting the sample that has the largest minimum distance from those already chosen, ensuring a uniform distribution in both x- and y-spaces [27].

For PLSR, 10 latent variables were chosen to maximize the covariance between the NIR spectra and the Urea-N reference values; this selection was based on observing a clear plateau in the explained covariance curve of the calibration set. The calibration models were built and evaluated using only the calibration dataset, monitoring the RMSEC (root mean square error of calibration) as additional confirmation of the optimal number of components. No formal cross-validation was performed; instead, model robustness was assessed by comparing RMSEC trends and R^2^ stability across successive increments of latent variables.

This approach ensured a balance between model complexity and predictive performance, avoiding overfitting while capturing the key spectral–chemical relationships.

### 2.7. Accuracy Evaluation of the Model

The calibration dataset provided the basis for the generation of regression models, which were independently verified using the subsequent dataset. The model outputs were evaluated using the R^2^, root mean square error (RMSE), residual prediction deviation (RPD), and root mean square error of prediction (RMSEP) [28], as defined in Equations (2), (3), (4), and (5), respectively.

(2) $$R^{2}=\;\frac{\sum_{i}^{n}{\left({\hat{y}}_{i}-y_{i}\right)}^{2}}{\sum_{i}^{n}{\left(y_{i}-\bar{y}\right)}^{2}},$$

(3) $$RMSE=\;\sqrt{\frac{\sum_{i}^{n}{\left({\hat{y}}_{i}-y_{i}\right)}^{2}}{n}}\;(\mathrm{identification}\;\mathrm{set}),$$

(4) $$RMSEP=\;\sqrt{\frac{\sum_{i}^{n}{\left({\hat{y}}_{i}-y_{i}\right)}^{2}}{n}}\;(\mathrm{validation}\;\mathrm{set}),$$

(5) $$RPD=\;\frac{SD}{RMSEP},$$

where

${\hat{y}}_{i}:predicted\;value\;of\;i_{th}\;observation;$

${\bar{y}}_{i}\;:\;mean\;observed\;value;$

$y_{i}\;:\;actual\;value\;of\;i_{th}\;observation;$

n :number of samples;

SD :standard deviation;

RMSEP : root mean square error of prediction.

In the context of evaluating regression models, the R^2^ value is used to assess stability, with a higher R^2^ indicating a better fit to the data, while the RMSE assesses the capacity of a model, with a smaller RMSE value indicating better predictive performance. The same information is provided by RMSEP, but using only the validation set. In summary, a model with a high R^2^ and low RMSE demonstrates a better fit to the data.

The model’s estimation capacity was evaluated using the RPD, which is the ratio of the standard deviation of the measured values to the RMSEP on the validation set.

The model’s estimation ability is very good if RPD > 2 and poor if RPD < 1, making it impossible to estimate the nitrogen content in the soil in the latter case [29].

## 3. Results and Discussion

**Figure 7:**
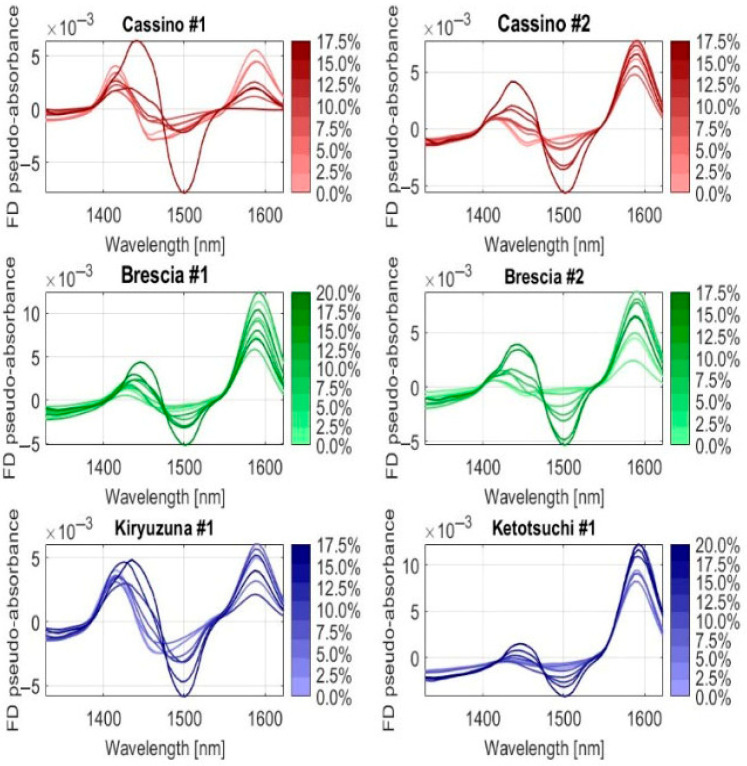
First derivative (FD) spectra of the six soils under different Urea-N concentrations (in %).

**Figure 8:**
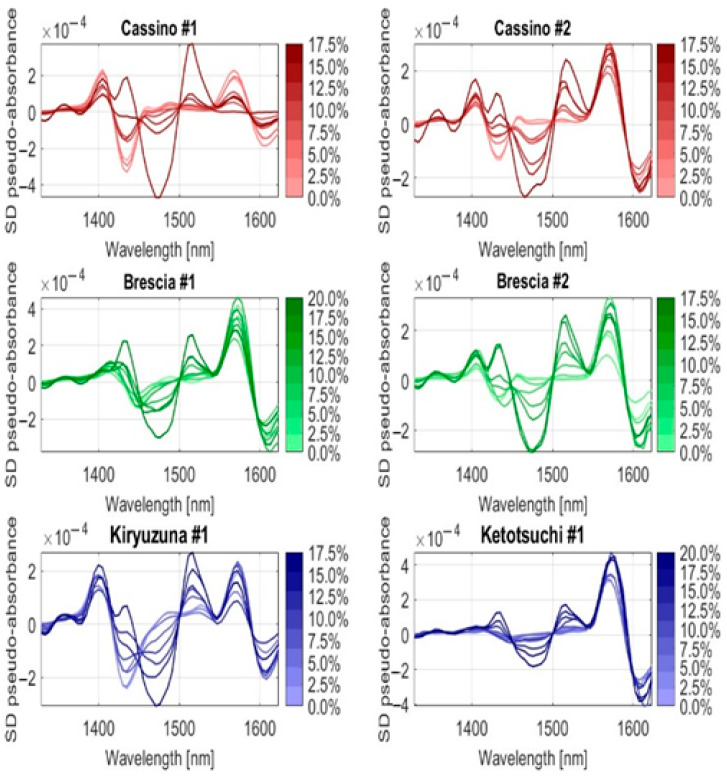
Second derivative (SD) spectra of the six soils under different Urea-N concentrations (in %).

To illustrate the locations of the characteristic wavelengths more clearly, the first and second derivatives of the average spectra of the six soils were calculated. In Figure 7 and Figure 8, the trends of the first and second derivatives are shown, respectively, as the percentage of Urea-N, so the nitrogen, varied from 0 to 20% by weight. Regardless of the morphology and composition of the six different soil types, they all responded with variations in the curve trends around the wavelengths of 1490 and 1520 nm, as expected and predicted according to Figure 2 above.

**Figure 9:**
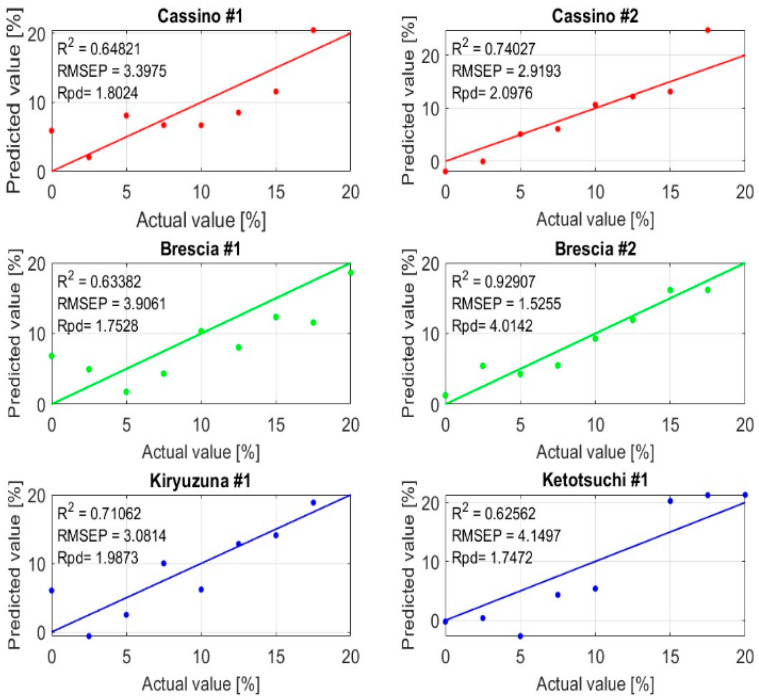
Scatter plot of predicted and measured Urea-N contents based on PLSR model after use of SG-FDT method.

The PLSR model was applied to the pre-processed data, and Table 4 and Table 5 show the obtained performances. In terms of calibration, the PLSR model performed well, achieving high R^2^ values (above 0.9) and low RMSE, with the SD-based pre-processing slightly outperforming the FD-based pre-processing in terms of accuracy. The very low bias in both cases suggests no systematic over- or under-estimation, indicating reliable performance during calibration. In terms of validation, the performance of the PLSR model slightly declined, with the FD-based pre-processing still outperforming the SD-based pre-processing in terms of R^2^, RMSEP, and RPD. The FD pre-processing approach showed moderate predictive ability (R^2^ = 0.77 and RPD = 2.06), while the SD pre-processing approach yielded weaker performance metrics (R^2^ = 0.65 and RPD = 1.77). The moderate (RPD = 2.06) estimation accuracy was likely due to the fact that the selected sensitive bands were not the most suitable for processing soil spectra. Figure 9 shows the scatter plot of correlations between the predicted and measured values of Urea-N using the SG pre-treatment method and first derivative (FD) as a reference. The prediction of Urea-N content for Brescia #2 soil was very good (RPD = 4). For the Cassino #2, Brescia #1, and Kiryuzuna #1 soils, the R^2^ values were around 0.7 (RPD of around 2), indicating a slightly lower level of prediction efficiency compared to Brescia #2. Cassino #1 and Ketotsuchi #1 seemed to exhibit some limitations in predicting Urea-N using a reduced spectral band (i.e., 1100–1700 nm), with RPD values around 1.5.

Another method used to evaluate performance in this study involved analyzing the percentage prediction error. Table 6 reports the minimum, maximum, and mean values of the prediction error for each soil type. As shown, the mean error is consistently close to zero across all soil types, highlighting the effectiveness of the PLSR algorithm in minimizing overall bias. The maximum and minimum values, on the other hand, represent the worst-case deviations observed within individual samples of each soil, indicating the range of prediction variability.

Expanding the band to longer wavelengths could potentially improve prediction performance; however, the objective of this study was to extract a key parameter related to soil fertility, nitrogen, by developing a prediction process that incorporates a wide variety of morphologically distinct soils to enhance generalizability. To this end, a lower-cost spectrometer was intentionally chosen to evaluate whether high-quality results could still be achieved with reduced instrumentation costs. When comparing some studies presented in the previous literature related to NIR techniques for assessing soil fertility (e.g., SOM), it seems that it is possible to confirm that the objective of the present research has been achieved. In fact, some studies have relied on NIR techniques for SOM extraction, but focused on a limited number of morphologically similar soils [10,11,12]. Others have used spectrometers with much broader bandwidths, reaching up to 2500 µm (compared to the 600 µm range used in this study, ranging from 1100 nm to 1700 nm), resulting in significantly higher costs than the solutions proposed here [30,31,32]. To demonstrate the validity of this work, Table 7 below collates data from several articles and compares them to ours.

This study focused on working with six distinct soils, which were morphologically very different and came from two different hemispheres. In the studies listed above, the soils were always from the same hemisphere and, in most cases, the number of different soils was lower. Even when up to ten soils [16] were used, they were collected from the same province, suggesting a similar morphology. The RMSEP value obtained is comparable to those reported in other works, even though it was based on a narrower spectral range. This makes our approach more efficient in terms of the cost-to-result ratio. In one study [14], better results (0.014%) were achieved using a different machine learning algorithm, but only one type of soil was considered there—meaning the method cannot be considered generalizable. In all the abovementioned studies, the way in which the results are reported is rather unclear. Only the last article (which focuses on comparing different machine learning techniques) clearly states the performance metrics. For example, in the first paper [30], the number of samples must be inferred from the text, as it is not explicitly stated. It is necessary to take into account that PLSR is a multivariate statistical method that works well when there are many variables and few observations. Since it is based on linear regression, using a very large number of samples increases the risk of overfitting. In fact, even in articles using many samples, the accuracy does not improve significantly.

The classification of a system as online or offline, as shown in Table 7, was inferred from the text, and refers to the entire spectral acquisition system, including the data collection, processing, and usage mode. The computational time is considered to be low when filters such as the Savitzky–Golay filter or linear statistical techniques like PCA and PLSR are used. On the other hand, methods like Random Forest usually require a longer processing time. The cost estimate is mainly based on the spectrometer: instruments capable of capturing a wider range of wavelengths are naturally more expensive.

## 4. Conclusions

This study evaluated the potential of NIR reflectance spectroscopy combined with chemometric techniques for predicting urea-nitrogen (Urea-N) levels across six distinct agricultural soil types. Among the tested pre-processing strategies, first-derivative (FD) and second-derivative (SD) transformations showed the best results, with the FD-based PLSR model achieving the highest predictive performance (R^2^ = 0.77, RPD = 2.06) during external validation. The analysis highlighted the spectral region around 1500 nm as particularly relevant for Urea-N estimation, corresponding to strong absorption features. While the calibration results were robust, a moderate reduction in performance during validation revealed some limitations in generalizability. These could be addressed by refining the spectral range, improving band selection, or incorporating more advanced model validation and regularization techniques. Overall, the proposed method enables rapid, non-destructive estimation of soil nitrogen content, offering a valuable tool for precision agriculture. Its ability to handle diverse soil types suggests strong potential for wider application in soil monitoring frameworks. Future work should explore broader soil datasets, integrate complementary machine learning approaches, and evaluate the performance of the system under real field conditions. Such developments would enhance the robustness and scalability of this approach, supporting more sustainable and data-driven soil management practices.

## Figures and Tables

**Table 1:** Area of origin and types of soil textures.

Soil Type	Country	Coordinate or Commercial Product ID	Soil Texture
Cassino #1	Sant’Angelo in Theodice, Cassino, South of Italy	41.45° N, 13.83° E	Clayey and sandy
Cassino #2	Caira, Cassino, South of Italy	41.530° N, 13.81° E	Clayey and sandy
Brescia #1	Montichiari, Brescia, North of Italy	45.41° N, 10.41° E	Sandy loam and alluvial
Brescia #2	Mompiano, Brescia, North of Italy	45.56° N, 10.23° E	Sandy loam and alluvial
Kiryuzuna #1	Northern island of Hokkaido, Japan	Bonsaischule Wenddorf DE-KIRYU-02	Volcanic
Ketotsuchi #1	Northern island of Honsu, Japan	Crespi Bonsai A518/02	Volcanic

**Table 2:** Urea-N contents in the six soil samples.

Soil Type	Content of Urea-N	Uncertainty	Number of Samples
	[%]	[%]	
Cassino #1	0, 2.5, 5, 7.5, 10, 12.5, 15, 17.5	0.17	8
Cassino #2	0, 2.5, 5, 7.5, 10, 12.5, 15, 17.5	0.17	8
Brescia #1	0, 2.5, 5, 7.5, 10, 12.5, 15, 17.5, 20	0.17	9
Brescia #2	0, 2.5, 5, 7.5, 10, 12.5, 15, 17.5	0.17	8
Kiryuzuna #1	0, 2.5, 5, 7.5, 10, 12.5, 15, 17.5	0.17	8
Ketotsuchi #1	0, 2.5, 5, 7.5, 10, 12.5, 15, 17.5, 20	0.17	9
	Total number of samples		50

**Table 3:** Composition of soil samples.

Soil Type	pH	Electrical Conductivity @ 20 °C	Available Nitrogen	Available Potassium	Available Phosphorous	SOM
		[µS/cm]	[g/kg]	[mg/kg]	[mg/kg]	[g/kg]
Cassino #1	7.5	414	1.3	2790	276	53
Cassino #2	7.7	852	0.1	4420	869	<1
Brescia #1	5.5	1710	1.1	13,900	968	116
Brescia #2	7.9	1900	4.4	4740	717	131
Kiryuzuna #1	6.0	93	2.6	123	218	<1
Ketotsuchi #1	5.1	2230	19.2	670	340	>207

**Table 4:** Calibration results of PLSR model using FD and SD spectra as pre-processing techniques.

Soil Attribute	Method	Calibration		
		R^2^	RMSE	BIAS
Urea-N [%]	1300–1650 nm FD and PCA	0.9	1.70	−4.5 × 10^−15^
Urea-N [%]	1300–1650 nm SD and PCA	0.93	1.53	−1.6 × 10^−15^

**Table 5:** Validation results of PLSR model using FD and SD spectra as pre-processing techniques.

Soil Attribute	Method	Calibration			
		R^2^	RMSEP	BIAS	RPD
Urea-N [%]	1300–1650 nm FD and PCA	0.77	4.36	−2.9	2.06
Urea-N [%]	1300–1650 nm SD and PCA	0.65	4.69	−1.62	1.77

**Table 6:** The max, min, and mean values of the prediction error for the six soils.

Soils	Prediction Error [%]		
	Min	Mean	Max
Cassino #1	−5.89	−1.33 × 10^−15^	3.98
Cassino #2	−7.23	−1.22 × 10^−15^	2.53
Brescia #1	−6.82	1.25	5.94
Brescia #2	−2.94	−1.11 × 10^−16^	2.00
Kiryuzuna #1	−6.05	7.77 × 10^−16^	3.79
Ketotsuchi #1	−5.24	0.94	7.68

**Table 7:** Comparison between articles from literature.

Study (Reference)	No. of Different Soils	No. of Samples	Contaminant to Detect in Soil	Wavelengths Used	Machine Learning	Prediction Accuracy (RMSEP or RPD)	Online / Offline	Computational Time	Cost
Yin, Z.; Lei, T.; Yan, Q.; Chen, Z.; Dong, Y. A near-infrared reflectance sensor for soil surface moisture measurement. Comput. Electron. Agric. 2013, 99, 101–107. https://doi.org/10.1016/j.compag.2013.08.029. [30]	4	~52	Soil moisture	900–1700 nm	SVR (reported as linear reg)	4.1%	Online	Low	High
Dhawale et al. (2015), Proximal soil sensing of soil texture and organic matter with a prototype portable mid-infrared spectrometer, *Eur. J. Soil Sci.* 66(4), 661–669. https://doi.org/10.1111/ejss.12226 [33]	4	60 (≈48 validation)	Sand, clay and SOM	2500–4000 nm	PLSR	10% (sand), 10% (clay), 2.3% (SOM)	Offline	Medium	High
Munawar A. A. et al. (2020), Calibration models database of near infrared spectroscopy to predict agricultural soil fertility properties, *Data Brief* 30, 105469. https://doi.org/10.1016/j.dib.2020.105469 [16]	10	40	N, P, K, pH	1000–2500 nm	PCR and PLSR	RPD > 2	Offline	Medium	High
Tan, B.; You, W.; Tian, S.; Xiao, T.; Wang, M.; Zheng, B.; Luo, L. Soil Nitrogen Content Detection Based on Near-Infrared Spectroscopy. Sensors 2022, 22, 8013. https://doi.org/10.3390/s22208013 [14]	1	43	N	900–1670 nm	Random Forest	RMSEP = 0.141 g/kg (~0.0141%)	Offline	High	High
Nawar et al. (2016), Estimating the soil clay content and organic matter by means of different calibration methods of vis-NIR diffuse reflectance spectroscopy, *Soil Tillage Res.* 155, 510–522. https://doi.org/10.1016/j.still.2015.07.021 [15]	5	75	SOM	350–2500 nm	OSC-PLSR, SNV-PLSR, MSC-PLSR	Best (MSC-PLSR): RMSEP = 4.8 % (RPD = 2.1)	Offline	Medium	High
**This article**	6	50	Urea-N	1100–1700 nm	SG + PCA + PLSR	RMSEP = 4.36% RPD = 2.06	Online	Low	low

## Data Availability

Data are contained within the article.
